# Autophagy counteracts instantaneous cell death during seasonal senescence of the fine roots and leaves in *Populus trichocarpa*

**DOI:** 10.1186/s12870-018-1439-6

**Published:** 2018-10-29

**Authors:** Natalia Wojciechowska, Katarzyna Marzec-Schmidt, Ewa M Kalemba, Aleksandra Zarzyńska-Nowak, Andrzej M Jagodziński, Agnieszka Bagniewska-Zadworna

**Affiliations:** 1 Department of General Botany, Institute of Experimental Biology, Faculty of Biology, Adam Mickiewicz University, Umultowska 89, 61-614 Poznań, Poland; 2 Institute of Dendrology, Polish Academy of Sciences, Parkowa 5, 62-035 Kórnik, Poland; 3 Department of Virusology and Bacteriology, Institute of Plant Protection, Węgorka 20, 60-318 Poznań, Poland

**Keywords:** Autophagy, *ATG* genes, ATG8 protein, Senescence, Leaves, Fine roots

## Abstract

**Background:**

Senescence, despite its destructive character, is a process that is precisely-regulated. The control of senescence is required to achieve remobilization of resources, a principle aspect of senescence. Remobilization allows plants to recapture valuable resources that would otherwise be lost to the environment with the senescing organ. Autophagy is one of the critical processes that is switched on during senescence. This evolutionarily conserved process plays dual, antagonistic roles. On the one hand, it counteracts instantaneous cell death and allows the process of remobilization to be set in motion, while on the other hand, it participates in the degradation of cellular components. Autophagy has been demonstrated to occur in many plant species during the senescence of leaves and flower petals. Little is known, however, about the senescence process in other ephemeral organs, such as fine roots, whose lifespan is also relatively short. We hypothesized that, like the case of seasonal leaf senescence, autophagy also plays a role in the senescence of fine roots, and that both processes are synchronized in their timing.

**Results:**

We evaluated which morphological and cytological symptoms are universal or unique in the senescence of fine roots and leaves. The results of our study confirmed that autophagy plays a key role in the senescence of fine roots, and is associated also with the process of cellular components degradation. In both organs, structures related to autophagy were observed, such as autophagic bodies and autophagosomes. The role of autophagy in the senescence of these plant organs was further confirmed by an analysis of *ATG* gene expression and protein detection.

**Conclusions:**

The present study is the first one to examine molecular mechanisms associated with the senescence of fine roots, and provide evidence that can be used to determine whether senescence of fine roots can be treated as another example of developmentally programmed cell death (dPCD). Our results indicate that there is a strong similarity between the senescence of fine roots and other ephemeral organs, suggesting that this process occurs by the same autophagy-related mechanisms in all plant ephemeral organs.

**Electronic supplementary material:**

The online version of this article (10.1186/s12870-018-1439-6) contains supplementary material, which is available to authorized users.

## Background

Senescence, as the final, inevitable stage of development before death, can occur in a select group of cells, tissues, organs, or even an entire plant. Seasonal senescence of organs is an adaptation that allows plants to adapt to a yearly change in environmental conditions. Regardless of the reason, senescence is a precisely regulated process that follows well-defined steps, clearly reflected by distinct physiological, cytological, and transcriptomic events [1, 2]. The precise control of senescence is necessary to allow the process of remobilization to occur, which is the main goal of prolonged senescence instead of rapid death [3]. During senescence, the degradation of cellular components is accelerated. The remobilization process allows those degraded components, that are still valuable for plants, to be transformed into forms that can be transported in the phloem and relocated to other parts of the plant e.g. to developing seeds or other storage organs [4–8]. There is also a body of evidence which demonstrates that autophagy plays a significant role in nutrient recycling during the senescence of plant organs [9–12].

Autophagy is an evolutionarily conserved, intracellular pathway in eukaryotic cells for the massive degradation of cytoplasmic components in a lytic compartment within cells [13]. It is responsible for the turnover of cytoplasm [14], scavenging of unnecessary cellular components [15], formation of some tissues [16–18], and biotic [19–23] and abiotic stress responses [24–28]. Thus, autophagy helps to preserve cell homeostasis. Microscopic observations of cells can distinguish three types of autophagy: micro-, macro-, and mega-autophagy [12, 29, 30]. During microautophagy, a small fragment of sequestered cytoplasmic constituents is incorporated into the vacuole by invagination of the tonoplast membrane [14, 31]. In macroautophagy, cellular material, or even entire organelles, intended for degradation are encapsulated in double-membrane vesicles called autophagosomes which are then transported to the vacuole. After fusion of the autophagosome and tonoplast membranes, the cytoplasmic cargo, contained a single membrane vesicle structure (autophagic body) is delivered into the vacuolar lumen [31]. Mega-autophagy, the third type of autophagy, begins with an intensive synthesis of hydrolytic enzymes, which results in enlarged vacuoles and increased tonoplast permeability. Finally, when the tonoplast is ruptured, the protoplast of the cell becomes acidified which leads to cell death [31].

The first evidence that autophagy plays a significant role in the controlled senescence of plant organs came from microscopic studies of senescing leaves of *Triticum aestivum*. Wittenbach et al. [32] observed that whole chloroplasts were present in the central vacuole which was filled with lytic hydrolases. In senescing petals of *Ipomoea purpurea* [33] and *Dianthus caryophyllus* [34], numerous vesicles containing fragments of degraded protoplast were observed. Similarly, in senescing fine roots of *Populus trichocarpa*, numerous autophagy-related structures have been observed [29]. As molecular tools developed, a plethora of mechanisms associated with autophagy were reported. In genetic screens of *Saccharomyces cerevisiae* for autophagy-defective yeast mutants, a number of *ATG* (*A*u*T*opha*G*y) genes required for autophagy were identified as being indispensable for the formation of autophagosomes during macroautophagy [13, 35]. The *ATG* genes and their protein products are also highly conserved in plants [14] and their occurrence and activity have been described in detail in *Arabidopsis* [36–38], rice [39], and maize [40]. The central core of autophagy machinery, which is necessary for autophagosome assembly, consists of 18 ATG proteins. These proteins can be divided into four groups based on their function: *(1)* the ATG1 protein kinase complex, which is necessary for induction and coordination of autophagy; *(2)* the PI3 kinase complex that is involved in the recruitment of the ATG18–ATG2 complex to PI3P in the autophagic membrane through an interaction between ATG18 and PI3P *(3)* the ATG9 complex which plays a role in delivering lipids to the pre-autophagosomal structure, and *(4)* two ubiquitination-like systems involved in elongation and enclosure steps during autophagosome formation (ATG12, ATG8) [41]. Analyses of gene expression indicated a significant increase in the expression of *ATG* genes during the senescence of leaves and flower petals [12, 42–44]. A significant role of autophagy in the senescence process was also confirmed in studies utilizing *Arabidopsis* mutants that displayed early and fast leaf senescence phenotypes [9]. In that study, the authors also indicated an intriguing role for autophagy in the remobilization process. The *atg* mutants are characterized by hypersensitivity to nitrogen, reduced seed production, and inhibition in the formation of Rubisco-containing bodies (RCB) [9]. Similar to leaves and flower petals, most fine roots, in contrast to pioneer roots, are short-lived [45]. Despite all the information that has been forthcoming on senescence, autophagy, and remobilization in leaves and flower petals, a similar level of understanding of the process of senescence in fine roots is lacking.

The most recent classification scheme classifies fine roots as first, second, and third order roots with a diameter < 2 mm [46]. They are characterized by a lack of secondary structure, the frequent presence of mycorrhizae, and a high surface to weight ratio [46]. These properties make them efficient in the absorption of water and minerals from the soil [47]. Fine roots, similar to leaves and flower petals, senesce and die after performing crucial functions that support plant growth and development. Root senescence and death have received a great deal of research interest over many years due to the importance of roots as a component of soil biomass and their effect on biological processes in forest ecosystems. The annual biomass production of fine roots is equal to or greater than the biomass of leaves, thus, the senescence and death of fine roots represent an important aspect of the cycling of chemical elements [48, 49].

In the present study, focus was placed on developing a more complete understanding of the process of fine root senescence relative to the same process in leaves. Despite the number of published root studies, few overall generalizations pertaining to the senescence process in roots have been established. This is perhaps principally because no conceptual framework exists for how root lifespan is constrained and controlled by cell or tissue physiology and genetics. While some theories to explain the control of fine root lifespan have been forwarded, very little data is available to evaluate these theories. Although this knowledge is crucial, obtaining high-quality data on this subject can be difficult and problematic. In the present study, we hypothesize that autophagy is an integral aspect of the senescence process in fine roots, as it is in seasonal leaf senescence, and that both processes are synchronized in their timing. We have conducted a significant amount of research to determine which morphological and cytological symptoms of root and leaf senescence are characteristic and either universal or unique to each organ. A molecular analysis of fine root senescence was also conducted, which provides the first evidence to support the premise that the senescence of fine roots can be seen as another example of developmentally programmed cell death (dPCD).

## Results

### Structure of senescing fine roots and leaves of *P. trichocarpa*

**Fig. 1:**
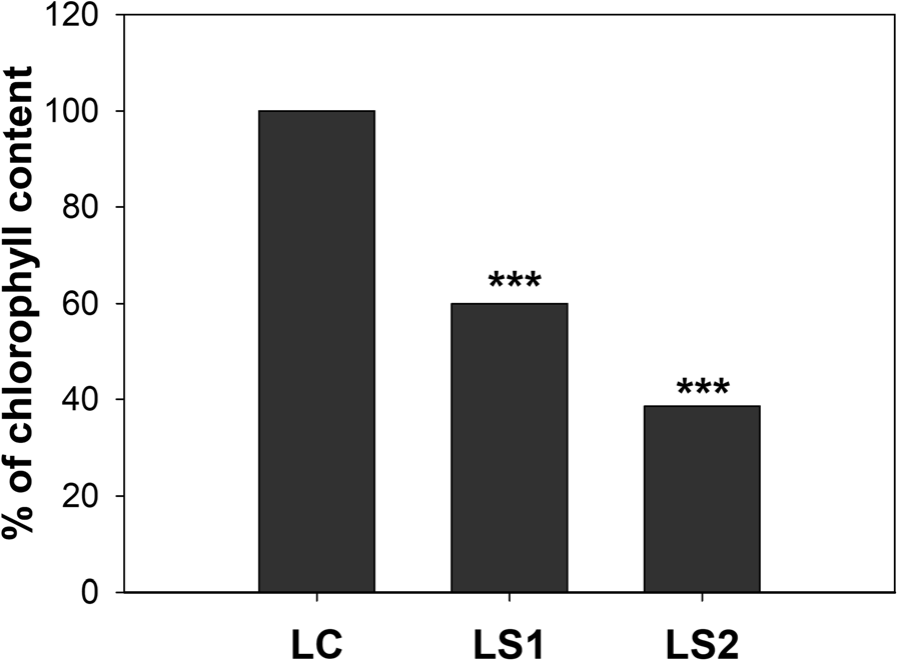
Changes in chlorophyll level in leaves during the growing season

Fine roots and leaves were systematically monitored during the growing season to detect the first visible/measurable symptoms of senescence. Therefore, several morphological, anatomical, and cytological features were identified. Chlorophyll levels were also measured in leaves (Fig. 1). After an analysis we classified the material studied into six groups and these groups were used as experimental variants in other studies. The six classified groups were designated as: green leaves - control (LC); two stages of senescing leaves - yellowing leaves (LS1) and yellow leaves (LS2); white fine roots - control (RC); and two stages of senescing roots - light brown roots (RS1) and dark brown roots (RS2).

### Morphological symptoms of senescence and cell viability in senescing organs

**Fig. 2:**
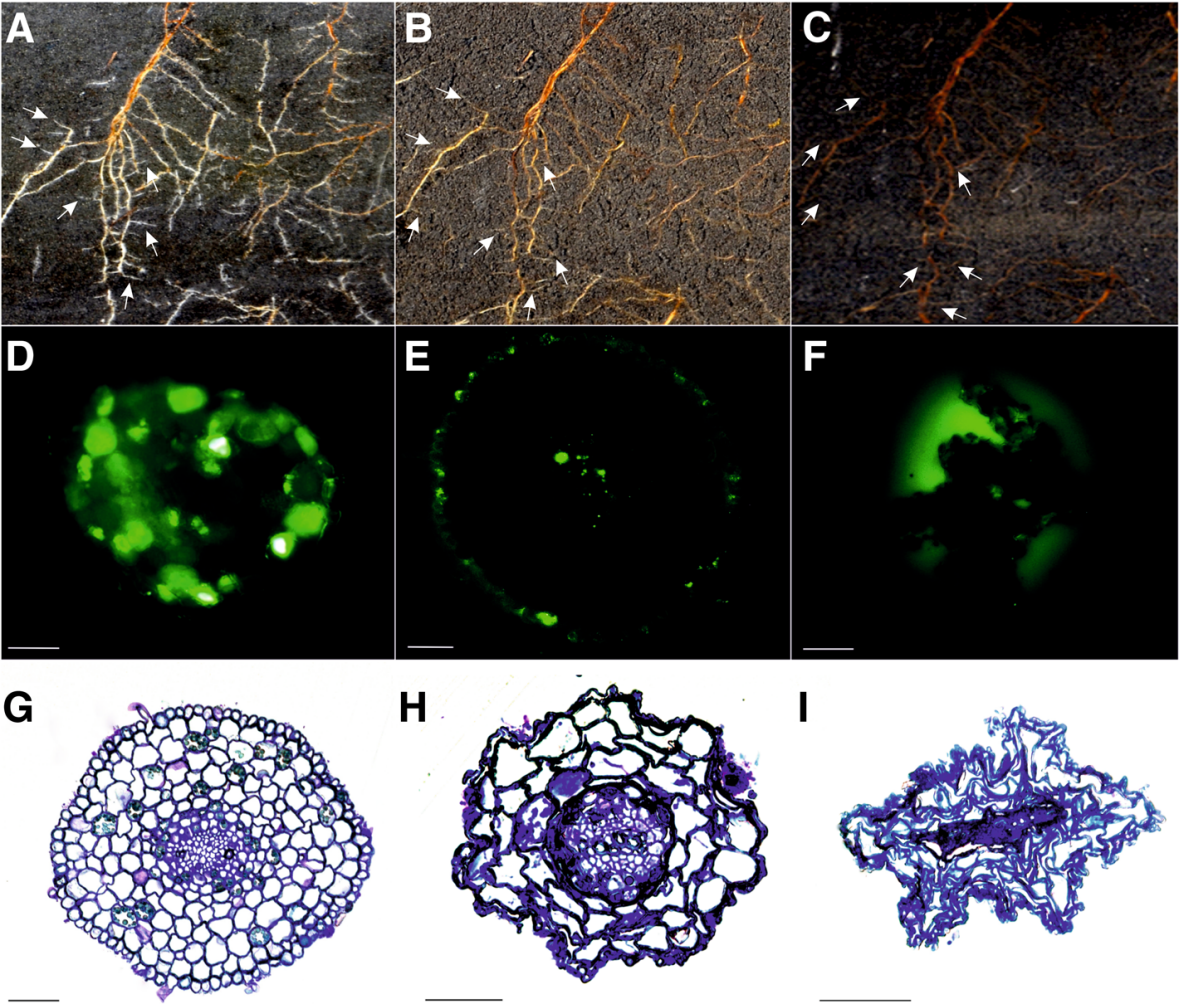
Senescence-related changes in fine roots (**a**-**c** – changes in morphology; **d**-**f** – changes in cell viability; **g**-**i** – changes in anatomy). Bars, 50 μm

**Fig. 3:**
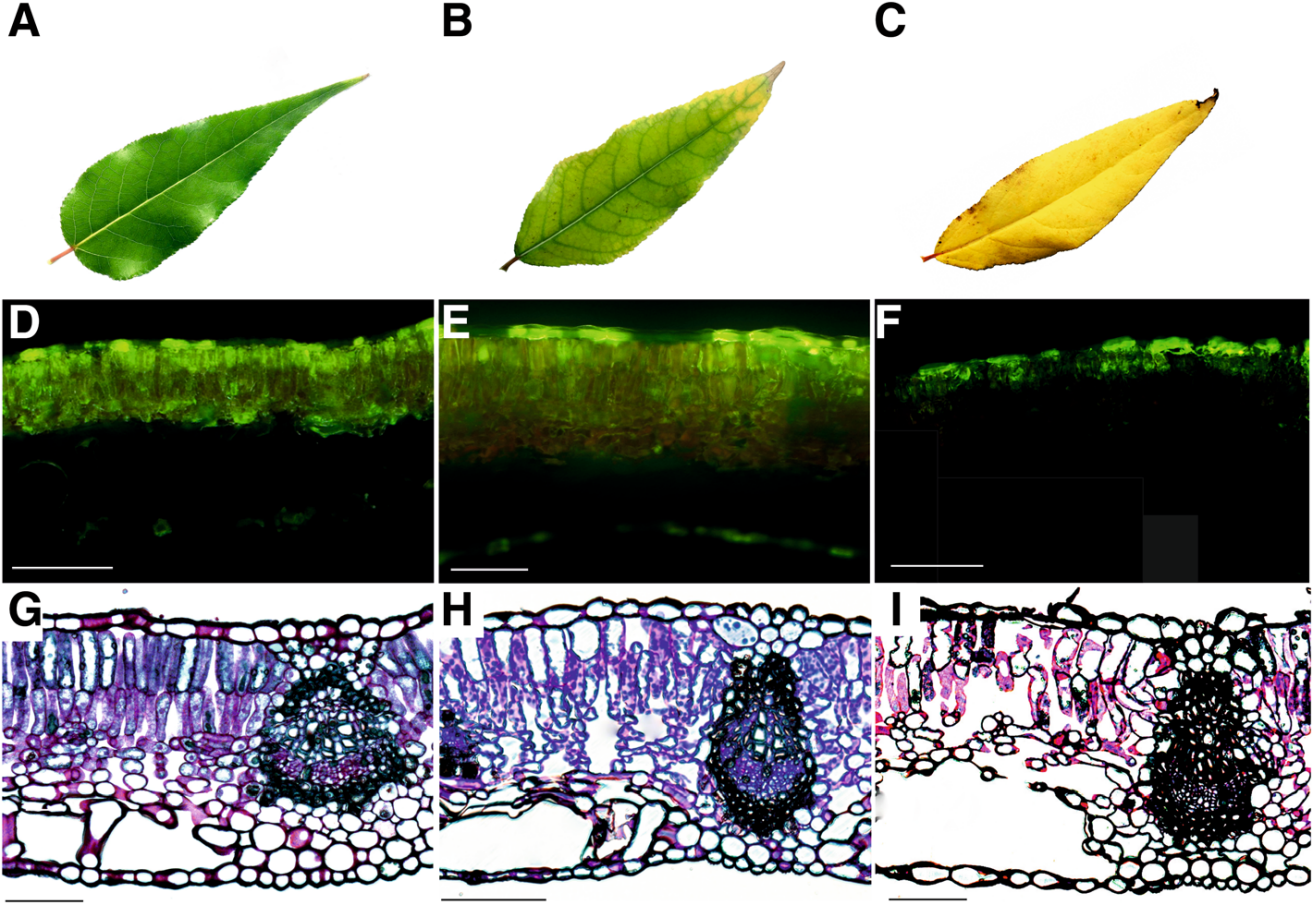
Senescence-related changes in leaves (**a-c** – changes in morphology; **d-f** – changes in cell viability; **g**-**i** – changes in anatomy). Bars, 100 μm

The pigmentation of both fine roots and leaves changed as the senescence process progressed (Fig. 2a-c; Fig. 3a-c). Fine roots changed in color from white to light brown to dark brown or black. A significant shrinkage in dark brown and black roots was also observed (Fig. 2b, c, h, i). Color changes in leaves were associated with decreases in chlorophyll content (Fig. 1; Fig. 3a-c). A viability assay was conducted to determine if the changes in color were associated with a loss of cell viability in leaves and fine root tissues. A fluorescent signal was observed in the majority of cells of white fine roots (RC) and green leaves (LC) (Fig. 2d; Fig. 3d); indicating a high level of cell viability. The number of cells with a fluorescent signal in the light brown roots (RS1) and yellowing leaves (LS1), however, decreased relative to the signal levels in control samples (Fig. 2e; Fig. 3e). Lastly, the fluorescent signal in dark brown roots (RS2) and yellow leaves (LS2) was very low and was not present in many of the analyzed sections of tissues (Fig. 2f; Fig. 3f).

### Anatomical characteristics of senescence

**Fig. 4:**
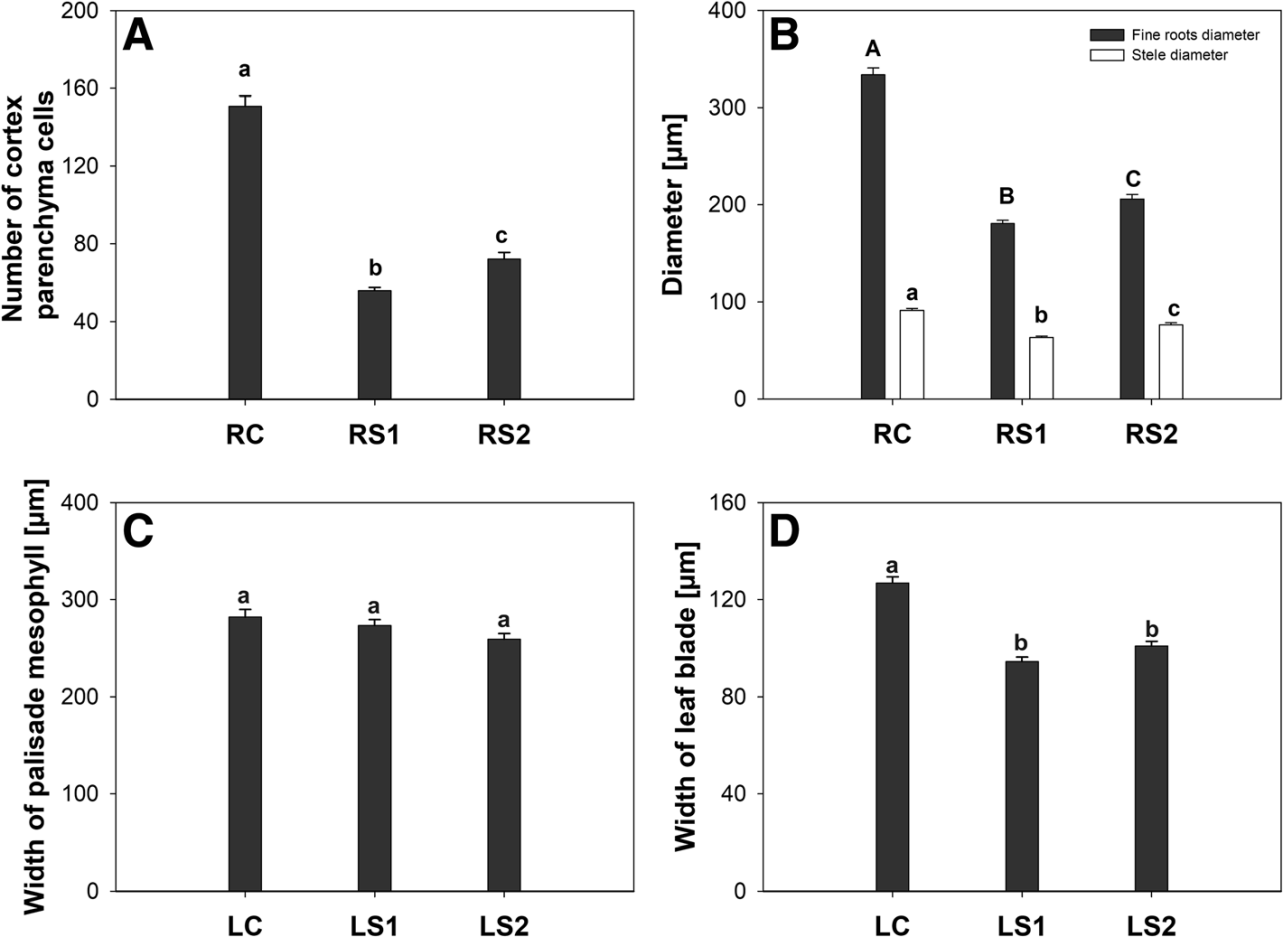
Changes in the structure of fine roots and leaves in relationship to the senescence process. **a** – Number of cortical parenchyma cells per section of fine roots. **b** – Changes in the diameter of roots and the stele during senescence. **c** – Width of the palisade mesophyll in leaves of *P. trichocarpa*. **d** – Width of the leaf lamina in *P. trichocarpa*. Bars sharing the same letter are not significantly different (*P* = 0,05). Values represent the mean ± SE (standard error)

An anatomical analysis using light microscopy was conducted to identify anatomical changes that were characteristic of the senescence process in two organs (leaves and fine roots). Pronounced, progressive changes were observed in fine roots. At the beginning of the growing season, fine roots (RC) were white and their morphology was round and regular (Fig. 2g). Internally, their cells had the appearance of features reflective of full turgor without any evidence of damage. The layer of cortical parenchyma cells was characterized by the presence of a large number of cells (Fig. 4a) without any evident signs of senescence. In the next two sampling periods (October and November), an increasing number of senescing roots were harvested. The most apparent characteristic in senescing fine roots (RS1 and RS2) were changes in their shape. Due to the occurrence of folded cell walls in cortical parenchyma cells, the morphological shape of the fine roots was not consistently round and regular, as had been observed in the RC root samples (Fig. 2h, i). This was confirmed by diameter measurements where a statistically significant decrease was apparent in RS1 and RS2 fine roots, relative to RC fine roots (Fig. 4b). Furthermore, many of fine roots collected at the RS2 stage were already dead and their overall structure was completely destroyed (Fig. 2i).

In contrast to fine roots, anatomical symptoms of senescence in leaves were not as readily evident (Fig. 3g-i). Significant changes in the shape of mesophyll cells were not observed, but the number of mesophyll cells was significantly lower relative to the control leaves (Fig. 3g-i). Measurements did not show statistically significant differences in the width of palisade mesophyll, however, a decrease in the leaf width occurred during the senescence process (Fig. 4c, d).

### Cytological analyses of senescing fine roots and leaves

**Fig. 5:**
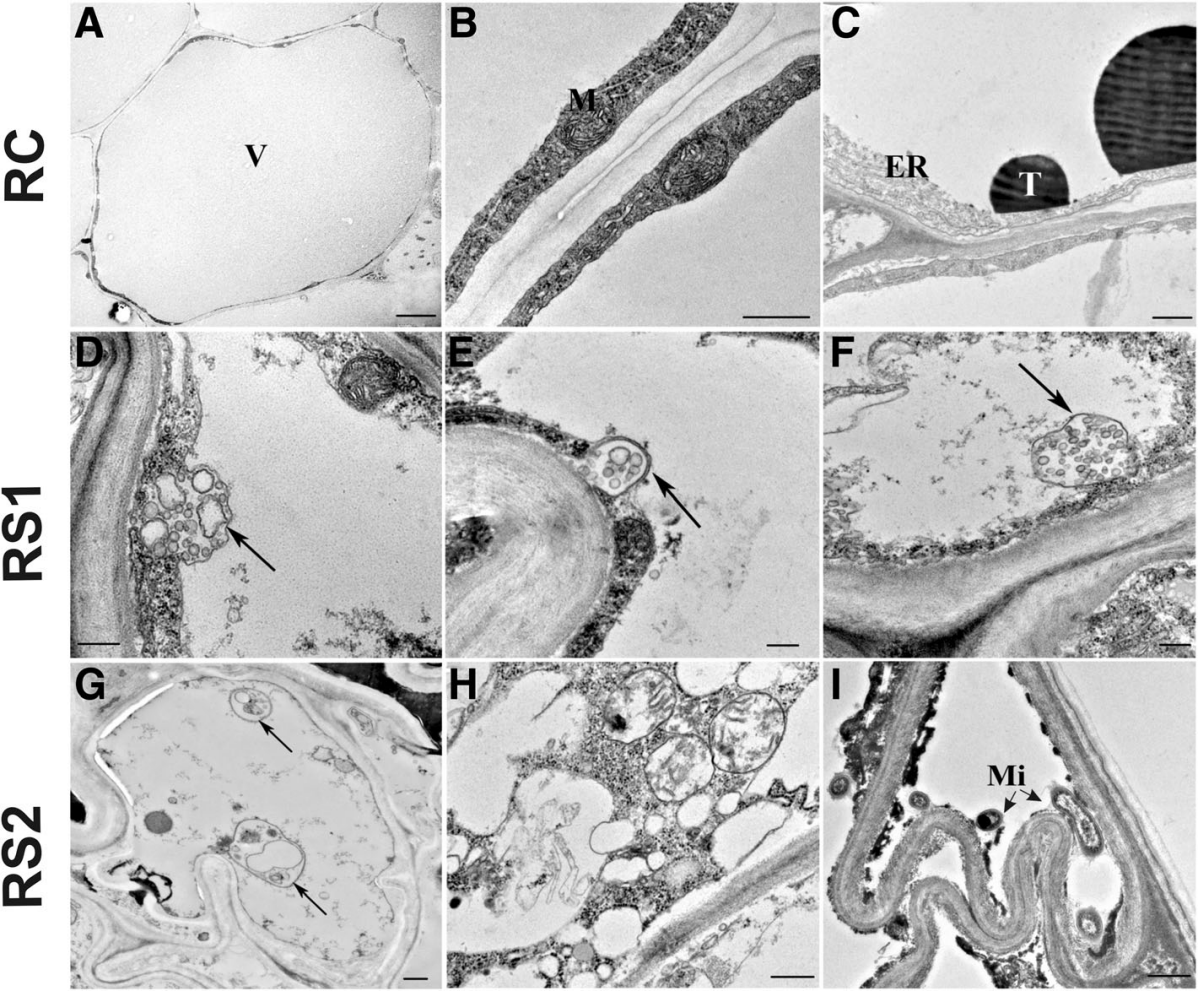
Changes in ultrastructure of cortical parenchyma cells in fine roots during the course of senescence. **a**-**c** - white fine roots - control (RC); **d**-**i** - two stages of senescing roots - light brown roots (RS1, **d**-**f**) and dark brown roots (RS2, **g**-**i**). Abbreviations: *V* vacuole, *ER* endoplasmic reticulum, *M* mitochondria, *T* tannins, *Mi* microorganism. Arrows indicate autophagy-related structures. Bars, 0,5 μm

**Fig. 6:**
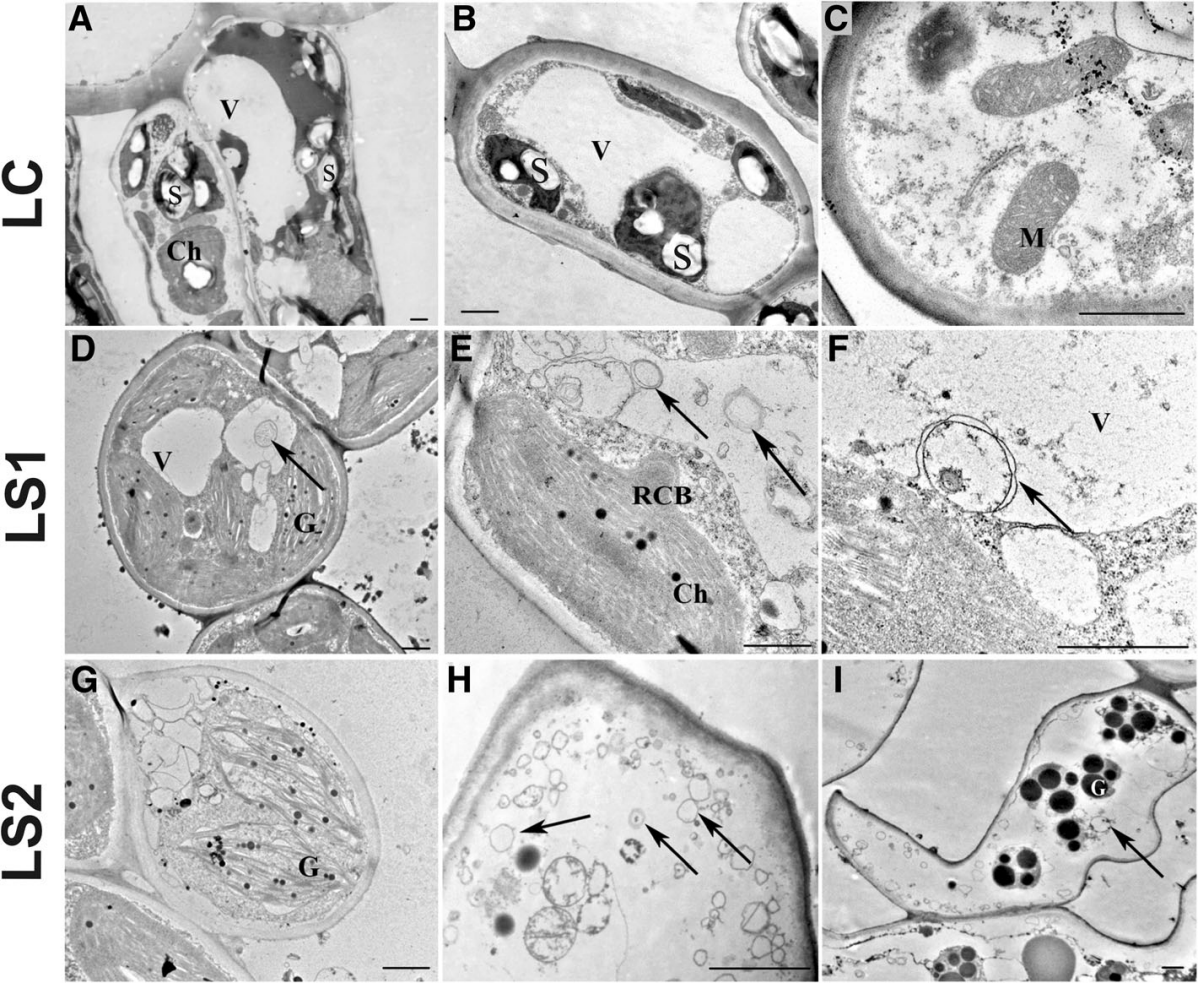
Changes in ultrastructure of palisade and spongy mesophyll leaf cells during the course of senescence. **a**-**c** - green leaves - control (LC); **d**-**i** - two stages of senescing leaves - yellowing leaves (LS1, **d**-**f**) and yellow leaves (LS2, **g**-**i**) Abbreviations: *V* vacuole, *S* starch, *M* mitochondria, *RCB* Rubisco containing bodies, *G* gerontoplast, *Ch* chloroplast. Arrows indicate autophagy-related structures. Bars, 1 μm

Based on the morphological and anatomical observations made of senescing leaf and fine root organs, cytological analyses focused on the cortical parenchyma cells of fine roots (Fig. 5) and the palisade and spongy mesophyll cells in leaves (Fig. 6). Cortical parenchyma cells in white, fine roots (RC) exhibited a regular shape with thin cell walls (Fig. 5a). A centrally located vacuole occupied most of the entire cell. The cytoplasm with its organelles was present as a thin band along the periphery of the cell wall (Fig. 5b). Tannins were observed in vacuoles of several cortical parenchyma cells, usually in close vicinity of the tonoplast (Fig. 5c). In contrast, evidence of senescence was readily observed in light brown (RS1) and dark brown (RS2) fine roots. The majority of cortical parenchyma cells in RS1 fine roots exhibited structures related to autophagy (Fig. 5d-f). Vesicles with cytoplasmic residues were observed in numerous cells. Those structures were similar to the vesicles present in cells undergoing microautophagy (Fig. 5d, e). Moreover, in RS2 cortical parenchyma cells, autophagic bodies inside vacuoles were also detected (Fig. 5g). Furthermore, the cell shape in the majority of RS2 cortical cells was more irregular than the oval shape of cells that were observed in RC and RS1 samples (Fig. 5h, i). Notably, cell walls were folded and the tonoplast was ruptured in cells that appeared to be in the last stage of senescence before dying. Furthermore, numerous microorganisms were observed in the external cortex of RS2 fine root samples (Fig. 5i).

Many changes in leaf ultrastructure related to the senescence process were also visible (Fig. 6). Control cells from LC were characterized by the presence of plenty organelles (mitochondria, endoplasmic reticulum and chloroplasts) with a normal appearance. Moreover, a significant number of starch granules were observed in both palisade (Fig. 6a) and spongy mesophyll (Fig. 6b) cells. In contrast, the appearance of the majority of the cells from yellowing leaves (LS1) was distinctly different (Fig. 6d-f). Among the different organelles, the first and most rapid alterations in response to senescence were observed in chloroplasts where the internal structure was greatly modified (Fig. 6d). Ultrastructural analysis revealed the disintegration of thylakoids, with a concomitant massive formation of plastoglobules that were mostly located between the thylakoids within the senescing chloroplasts (Fig. 6d). Moreover, spherical bodies separating themselves from chloroplasts were observed in several cells, including Rubisco-containing bodies (RCB) (Fig. 6e). Furthermore, several different autophagy-related structures were observed in the cytoplasm, including autophagic bodies in the vacuole lumen (Fig. 6d) and autophagosomes (Fig. 6e). Evidence of the formation of these structures was also observed, seen as the joining of several tubules and vesicles (Fig. 6f). Many cells in yellow leaves (LS2) exhibited more advanced senescence-related changes (Fig. 6g-i). The structure of chloroplasts was more visibly altered, an increasing number and size of plastoglobules were observed (Fig. 6g), as well as more distended thylakoids. Ruptured tonoplasts were observed in several cells, which resulted in the degradation of all cellular structures due to the acidification of the cytoplasm that occurred once the tonoplast was ruptured (Fig. 6h, i).

### Expression of *ATG* genes during senescence

**Fig. 7:**
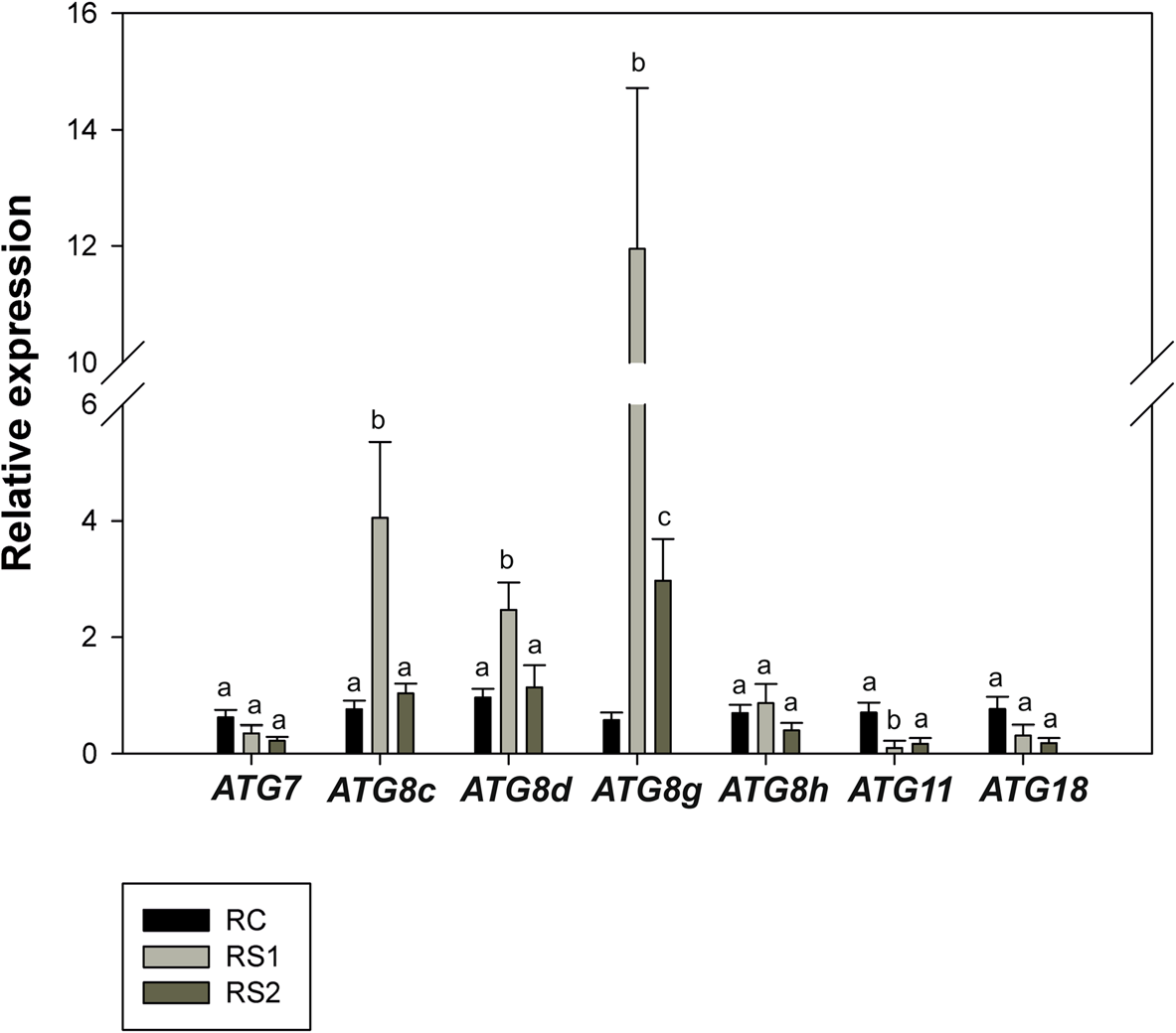
Relative expression of *ATG* genes in fine roots (RC – root control, RS1 –first stage of root senescence, RS2 –second stage of root senescence) of *Populus trichocarpa*. Bars sharing the same letter are not significantly different (*P* = 0,05). Values represent the mean ± SE (standard error)

**Fig. 8:**
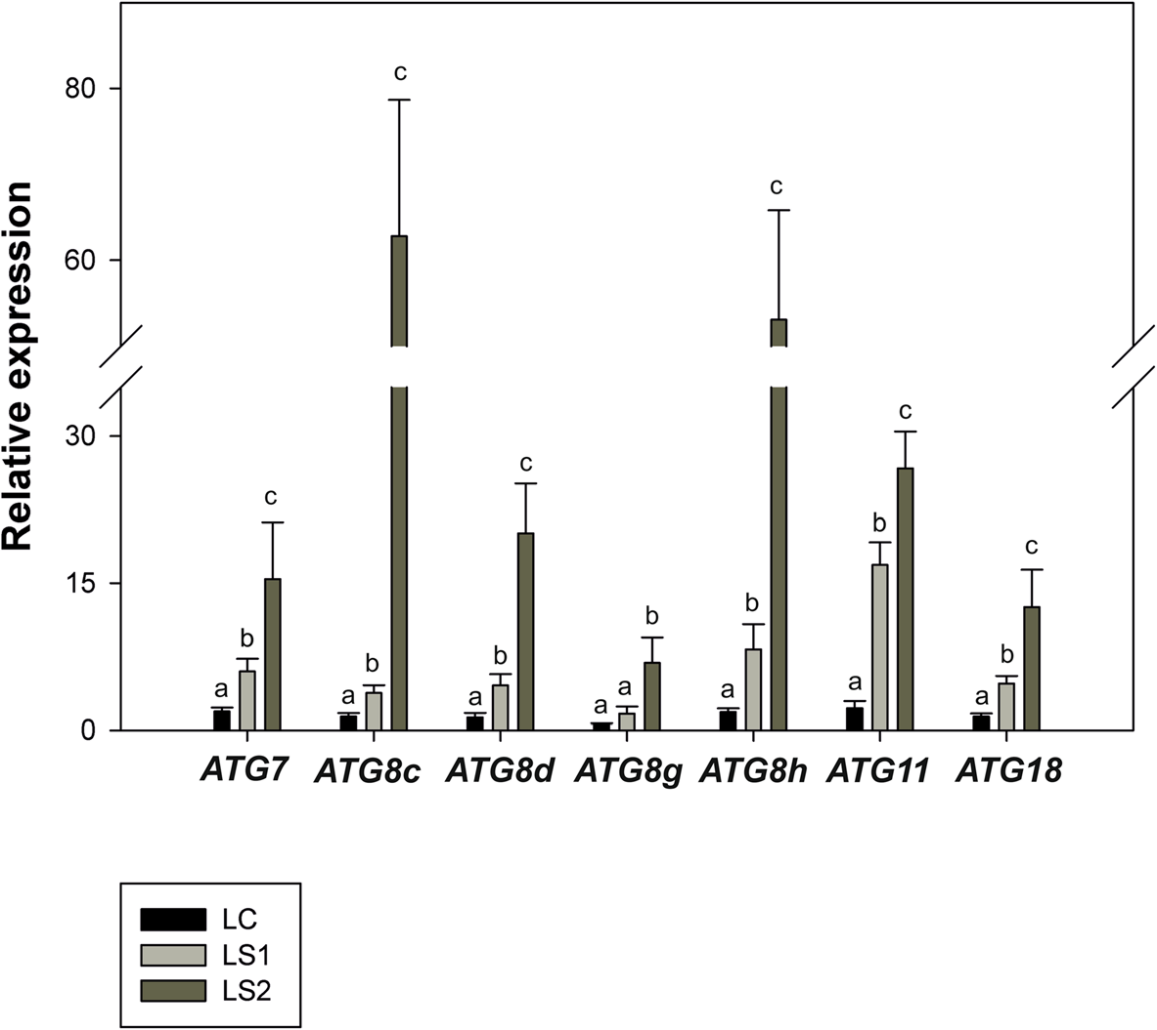
Relative expression of *ATG* genes in leaves (LC – leaf control, LS1 –first stage of leaf senescence, LS2 – second stage of leaf senescence) of *Populus trichocarpa*. Bars sharing the same letter are not significantly different (*P* = 0,05). Values represent the mean ± SE (standard error)

The analysis of *ATG* genes expression revealed significant differences in gene expression between control and senescing leaf and fine root tissues (LC vs LS and RC vs RS). The expression of *ATG7*, *ATG8c*, *ATG8d*, *ATG8g*, *ATG8h*, *ATG11*, and *ATG18* were examined (Fig.7; Fig. 8). Statistically significant changes in the expression of majority *ATG8* genes (*ATG8c*, *ATG8d*, *ATG8g*) were observed in fine roots (Fig. 7). Expression of all of these genes increased at the first stage (RS1) of senescence and then decreased in the second stage (RS2) of senescence (Fig. 7). In contrast, a slightly different pattern of expression was observed in leaf tissues. In contrast to roots, the expression of all of the examined *ATG* genes was upregulated in leaf tissues in both stages (LS1 and LS2) of senescence (Fig. 8). The largest increase in expression level was observed in the second stage (LS2) of senescence.

### Distribution and localization of ATG8 protein

**Fig. 9:**
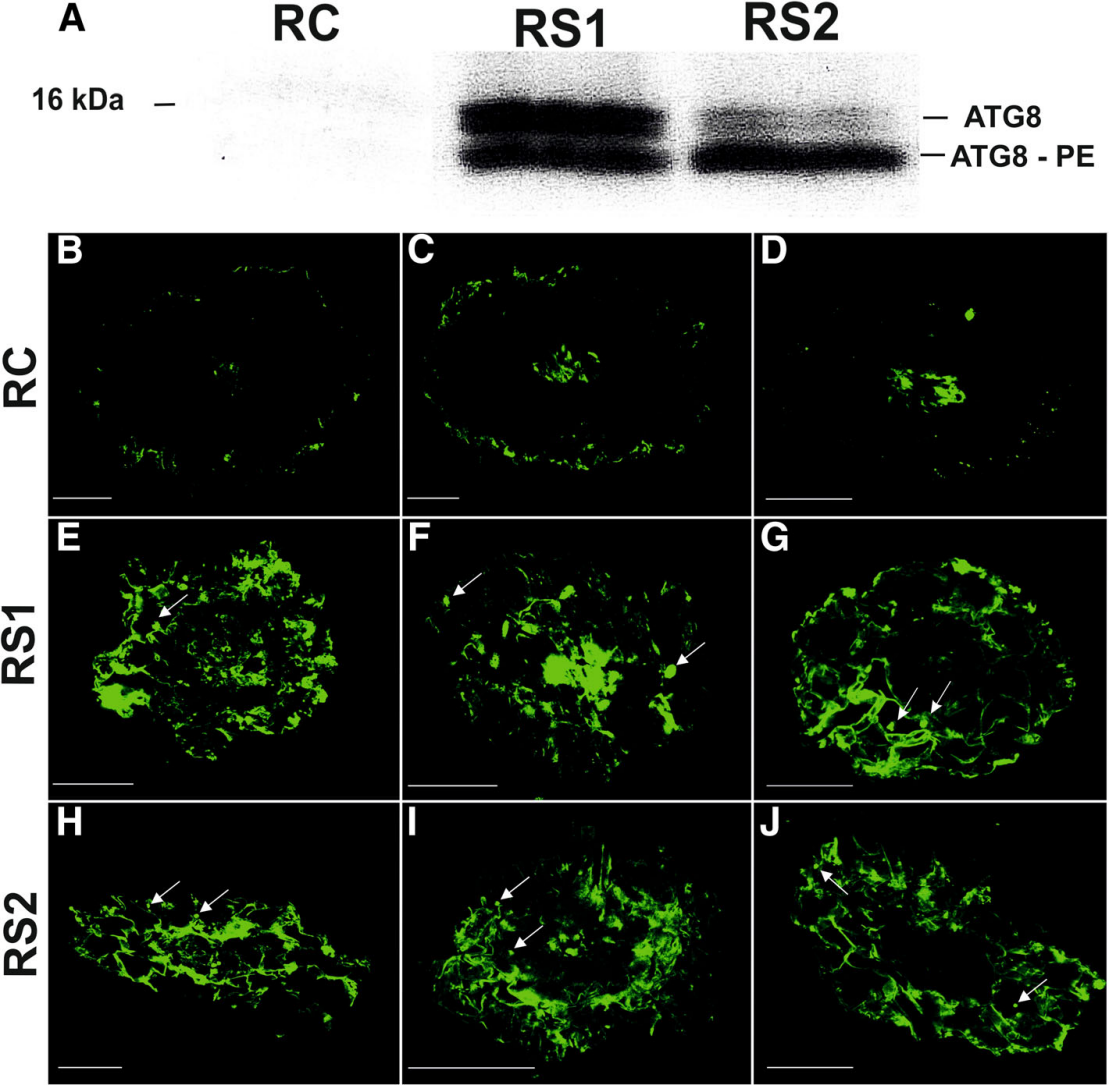
ATG8 protein levels (**a**) and the immunolocalization of ATG8 protein (**b-j**) in fine roots (RC – root control, RS1 – first stage of root senescence, RS2 – second stage of root senescence) during the growing season. Bars, 50 μm

**Fig. 10:**
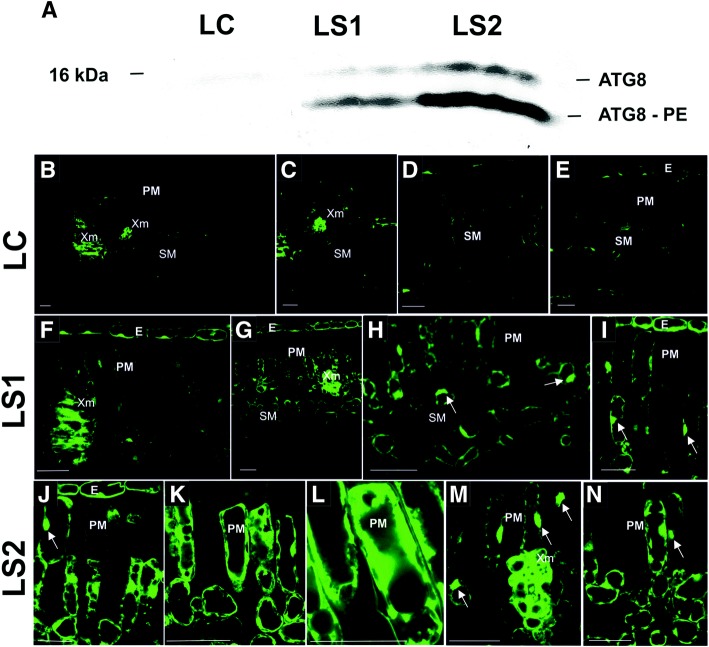
ATG8 protein levels (**a**) and the immunolocalization of ATG8 protein (**b-n**) in leaves (LC – leaf control, LS1 – first stage of leaf senescence, LS2 – second stage of leaf senescence) during the growing season. (Abbreviations: *Xm* xylem vessels, *PM* palisade mesophyll, *SM* spongy palisade, *E* epidermis). Bars, 25 μm

Based on the significantly increased expression of *ATG* genes in both roots and leaves, the amount and localization of ATG8 protein, which is necessary for appropriate autophagosome formation, was examined by immunoblot (Western blot) and immunolocalization analyses. ATG8 protein can be detected either as a protein conjugated to phosphatidylethanolamine (PE) on an autophagosomal membrane or as a free protein without PE. The level of ATG8 protein in both fine roots and leaves changed over the course of the growing season (Fig. 9a; Fig. 10a). Results indicated that the amount of ATG8 protein exhibited a similar pattern to changes in *ATG8* gene expression.

The level of ATG8 was relatively low in viable, white roots (RC) (Fig. 9a). ATG8 was located mainly in xylem tissues or cells of the rhizodermis (Fig. 9b-d). A significant increase in the level of ATG8 was observed in the first stage (RS1) of senescence when fine roots appeared brownish (Fig. 9a). Both forms of ATG8 (free and conjugated to PE) were detected. ATG8 was localized in the majority of cortex parenchyma cells and in xylem tissues (Fig. 9e-g). ATG8 was detected in the cytoplasm, near the cell wall, or more concentrated in spherical bodies (Fig. 9e-g, arrows). Subsequently, when roots became dark brown (RS2), a slight decrease in the level of ATG8 was observed in fine root tissues. ATG8 conjugated to PE was the main form observed in RS2 fine root cells. The level of free protein was clearly lower in RS2 than in RS1 fine root cells (Fig. 9a). The localization of the conjugated and free protein did not appear to significantly change between the RS1 and RS2 stages of senescence (Fig. 9h-j).

The level of the free form of ATG8, as well as the form in which ATG8 is conjugated to PE, was very low in green leaf (LC) tissues (Fig. 10a). A positive localization signal was mainly observed within the vascular bundle in xylem cells (Fig. 10b, c). In palisade and spongy mesophyll cells, ATG8 was localized in several cells but the signal level was relatively low (Fig. 10b-e). The level of ATG8 noticeably increased in yellowing leaves (LS1) and was mainly the form in which ATG8 is conjugated to PE (Fig. 10a). The signal was localized in epidermal cells, as well as the spongy and palisade mesophyll (Fig. 10f-i). ATG8 protein was mostly localized in spherical bodies (Fig. 10h, i, arrow) which were located in proximity to the cell wall. A significant level of localization also occurred in xylem vessels (Fig. 10f). A notable increase in the ATG8 level was observed in yellow leaves (LS2) (Fig. 10a). Microscopic analysis also revealed a strong localization of ATG protein in most cells (Fig. 10j-n). ATG8 protein was localized in cells of the epidermis, spongy and palisade mesophyll, and in xylem vessels. The distribution of ATG8 was similar in both the LS1 and LS2 stages of senescence, where it was concentrated in spherical bodies (Fig. 10j, m, n, arrow) but also dispersed in the cytoplasm (Fig. 10j-n).

## Discussion

In this work, we emphasize the universality of senescence, which occurs in all ephemeral organs, and further indicate that regardless of the organ being examined, some aspects of senescence are common to all aging processes. Despite copious research conducted on programmed cell death in plants, a detailed understanding of the mechanisms underlying autophagy, which occurs during the senescence of all organs and tissues, is still insufficient. A key question is whether common mechanisms can be identified that are responsible for senescence in different plant organs? While the very precisely controlled death of specific cells during early development has been well described, the process of senescence in plants is well described only for leaves, fruits, and flower petals. Although senescence also occurs in below ground plant organs, this process is barely understood in root systems due to the difficulty in harvesting of roots. In spite of, or perhaps because of, these limitations, the present study compared the natural senescence process that occurs in two different plant organs: leaves and fine roots. We were interested to determine whether organs that serve completely different functions and possess a completely different structure undergo senescence in a similar manner. Morphological, anatomical, cytological, and molecular characteristics were used to analyze this question.

Utilizing the FDA viability tests, both fine roots and leaves were confirmed to undergo a gradual decrease in cell viability, along with morphological symptoms of senescence. Moreover, the decrease in viability observed in leaves and fine roots was synchronized in its timing, indicating that senescence in these two organs is induced by the seasonal change in environmental conditions. Our results are consistent with those obtained by Comas et al. [50] and Bagniewska–Zadworna et al. [29], who investigated the senescence of roots in *Vitis labruscana* and *Populus trichocarpa,* respectively. These studies also observed a progressive decrease in cell viability during the senescence process. The morphological features that were observed in our study were associated with a change in the pigmentation of the senescing organs and a shrinkage of the entire organ. Indeed, the first noticeable similarity in the senescence of fine roots and leaves was a change in color. The change in leaf color, which results from the degradation of chlorophyll, has been often described in numerous plant species, including *Glycine max* and *Arabidopsis thaliana* [51], *Chenopodium quinoa* [52], *Gossypium hirsutum* [53]. Chlorophyll degradation during leaf senescence exposes carotenoids [54], and is the cause of the change in leaf color that occurs in autumn in deciduous trees. The color change of flower petals, another ephemeral organ, has also been documented in several plant species from different plant families, including *Ipomoea nil* [55, 56], *Nicotiana mutabilis* [57], *Antirrhinum majus* [58], *Argyranthemum frutescens* [58], and *Petunia hybrida* [59]. The senescing petals of *Hibiscus syriacus* become blueish when the ratio of flavonoids and anthocyanins changes and alters the pH of the cytoplasm [60].

Another morphological characteristic that occurs universally during the senescence of every ephemeral organ is shrinking and/or wilting [2]. In the present study, shrinkage was evident during the senescence of most fine roots of *Populus trichocarpa* in the second stage (RS2) of senescence. This observation is similar to the reports in earlier root studies [29, 50]. In contrast, a decrease was observed in the width of the leaf blades of *Populus trichocarpa* during leaf senescence. This may have been related to a loss in cell turgor, which makes the leaves appear withered.

A common sequence of events during the senescence of fine roots and leaves was also observed at an ultrastructural level. In both organs, the shape of cells became irregular and altered during the senescence process, which was in sharp contrast to the regular outline of cell shape observed at the beginning of the growth season. It is plausible that the change in cell shape may have been induced by an impairment of the cytoskeleton [61]. Early degradation of the lattice formed by cortical microtubules was reported to occur during both natural and dark-induced senescence of *Arabidopsis* leaves [61]. The expression of genes related to the cytoskeleton, such as α-, β-, and γ-tubulins, were also reported to be repressed during leaf senescence [42].

Another common feature of the senescence process appears to be the occurrence of autophagy, which has been observed to occur at the beginning of senescence, evidenced by the accumulation of a large number of vesicles in senescing cells. These vesicles most likely formed through micro and/or macroautophagy, as evidenced by their localization and appearance, which indicated vesicle formation. The formation of multiple vesicles by the fusion of several tubules was evidence of macroautophagy according to van Doorn and Papini [62]. Spherical bodies separated from chloroplast, and remaining Rubisco-containing bodies (RCB) were also observed in leaf cells in the present study. RCB bodies are double-membrane vesicles which contain chloroplast proteins such as Rubisco and Gln synthetase [63, 64]. The presence of numerous double membraned vesicles was also observed in senescing petals of *Ipomoea purpurea* [33] and *Dianthus caryophyllus* [34]. These observations indicate that autophagy plays an important functional role during the senescence of all ephemeral organs, where it is equally responsible for degradation of cellular components and the selective recycling and remobilization of chemical constituents.

Autophagy is a universal mechanism in cells that is responsible for the degradation of aberrant proteins and damaged organelles so that cellular homeostasis is maintained [65, 66]. Autophagy is typically accompanied by the process of programmed cell death (PCD). This relationship has been confirmed during various developmental events in plants, such as xylogenesis [67], anther development [16], tapetum degradation [16], and the hypersensitive response (HR) [21]. Similar mechanisms may regulate cell death during the senescence of leaves and flower petals [43, 55, 64, 68, 69]. A significant knowledge gap still exists, however, regarding the presence of autophagy in the senescence of fine roots and details of its functional role.

Similar to the senescence process in leaves and petals, autophagy in fine roots is also involved in the disintegration of membranes, and delivering unwanted cytoplasmic material, such as targeted proteins, carbohydrates, and lipids, to vacuoles for breakdown; thus replenishing the supply of nutrients needed for normal cell function. Therefore, autophagy often plays a dual antagonistic role as executioner and as a mediating, dilatory factor in senescence. Ultrastructural studies performed on senescing leaves and fine roots of *P. trichocarpa* in the present study provided many general observations. Many autophagy-related structures were observed in the cytoplasm and vacuole lumens of both leaf and fine root cells. To provide evidence supporting the origin of these vesicles and their association with autophagy in both organs, *ATG* gene expression and ATG protein levels were analyzed. The expression of the selected *ATG* genes increased in both leaf and fine root tissues during senescence. The highest increase in expression among the analyzed genes was observed for *ATG8* genes. ATG8, is a ubiquitin-like peptide tag which is necessary for formation of autophagosomes and is responsible for regulating their size [13, 70, 71]. ATG8 is conjugated to phosphatidylethanolamine (PE) on an autophagosomal membrane by a bond between the carboxyl-terminal glycine (Gly) of ATG8 and PE [12]. In various studies, ATG8 and its homologs (LC3 in mammals) was used as a reliable marker for the induction and progression of autophagy. Several *ATG8* genes have been identified in the plants [72]. The expression of *ATG8* genes observed in the present study in senescing organs (leaves and fine roots) of *P. trichocarpa* was slightly different in the two organs. In leaves, *ATG8c* and *ATG8h* exhibited the highest level of expression, while *ATG8g* was the most upregulated in fine roots. Tissue-specific expression of *ATG8* genes has also been observed in *Arabidopsis* [72]. During developmental and dark-induced senescence, *ATG8* expression has been reported to increase in leaves of *Arabidopsis thaliana* [42] and *Hordeum vulgare* [44], as well as in senescing petals of *Petunia hybrida* and *Impomea nil* [12, 65]. The involvement of autophagy in senescing fine roots was convincingly confirmed in our study by protein analysis, which indicated that the level of ATG8 protein significantly increased in senescing roots. ATG8 protein was localized in the cytoplasm and highly concentrated in specific, membrane bound structures. A similar observation was reported by Thompson et al. [72], who detected ATG8 fused with GFP in hypocotyl cells of young seedlings during N starvation.

Only a few studies can be identified where meaningful evidence of the dual role of autophagy during the senescence of plant organs has been provided. Although the dual role of autophagy as both a pro-survival and pro-death process was recently discussed [41], most studies have only focused on its role in the process of degradation. In the 1980s, electron microscopy provided visual evidence of chloroplast degradation and the presence of degraded chloroplast components in vacuoles [32]. Later, Ishida et al. [63] reported the accumulation of small bodies, which were designated as Rubisco containing bodies (RCB) based on their composition, in senescing leaves of *Triticum aestivum*. Plants constitutively expressing stroma-targeted GFP demonstrated that the accumulation of the GFP signal was localized in the vacuolar lumen of cells treated with concanamycin A, a drug that inhibits the degradation of autophagic bodies in the vacuole. Interestingly, RCB bodies were not observed in the cells of *atg5* mutants, suggesting that the autophagy-dependent process is responsible for the degradation of chloroplasts. A similar result was obtained with *Arabidopsis* mutants, *atg4a, atg4b-1*, which exhibit autophagy disorders and where RCB bodies were also not detected [73]. Degradation of chloroplasts by autophagy was unequivocally confirmed in studies where co-expressed stroma-targeted RFP and ATG8 fused with GFP colocalized in the vacuole of leaves [63, 74]. Autophagy plays the role of an executioner in the last stage of senescence process when increased permeability and eventual rupturing of the tonoplast membrane; resulting in the release of hydrolytic enzymes which cannibalize the protoplast and cause cell death [66]. Rupture of the tonoplast membrane represents the point-of-no-return and the described sequence of events has been observed in senescing leaves [75], flower petals [33], and fine roots [29].

Much less attention has been paid to the role of selective autophagy in the remobilization process [9–11]. Additionally, knowledge concerning the mechanisms and function of autophagy in nutrient availability and recycling in plants is less advanced for roots than it is for leaves. The first evidence for the role of autophagy in remobilization was provided in studies of *Arabidopsis* leaf senescence [9]. Genetic and molecular analyses utilizing mutants with impaired *ATG* genes help to document the biological function of autophagy in remobilization. Using wild-type (WT) and *atg* mutants of *A. thaliana* treated with 15NO_3_^−^, the level of 15 N was evaluated. Results indicated that remobilization was significantly lower in the *atg* mutants than in WT plants [9]. Interesting results regarding the relationship between autophagy and remobilization during senescence came from a study of maize *atg12* mutants [11]. This study demonstrated that 15 N remobilization to seeds was altered in *atg12* autophagy-defective mutants. Surprisingly, the relocation of nitrogen to newly-formed leaves was greater in the *atg12* autophagy-defective mutants as compared to WT. Remobilization of nutrients is also observed during the senescence of flower petals. Quantitative analysis of nitrogen in *Petunia hybrida* flowers demonstrated that the level of N changed before and after pollination-induced senescence in the examined parts of a flower. Nitrogen content decreased in petals and increased in the ovaries of pollinated flowers [12].

The role of autophagy in remobilization is also essential in fine roots. Fine roots are characterized by a short lifespan which typically does not exceed two years [76, 77]. In *Populus*, the life-span of fine roots is usually < 95 days [78]. Considering that the biomass of fine roots is equal to or greater than the biomass of leaves, remobilization is an important subject when discussing the cycling and recycling of chemical elements [48, 49]. Our current study indicated that the autophagy machinery is present and active in senescing fine roots, and suggests that substantial amounts of the elements stored in fine roots are remobilized to other parts of the plant. How large a portion is released to the soil may be also regulated by autophagy. Identifying the reason and underlying mechanism for the induction of autophagy and its biological function during the time period prior to the final death of fine roots will require additional studies.

## Conclusion

The senescence of plant organs, despite its destructive character, is a genetically controlled process that follows a well-defined sequence of events and is regulated by multiple pathways [2]. Cell viability is also essential for the initiation and progression of cell senescence. As long as a cell is viable, autophagic processes can be utilized to continue the process of degradation and remobilization in a controlled manner without crossing the point-of-no return and the final result, cell death. Our study comparing the senescence process in fine roots and leaves, helps to establish a cohesive model of the process of senescence in ephemeral organs. The combination of current and long-established information, clearly indicates that autophagy is a multifaceted system that plays a role in both the degradation of unwanted, unneeded cellular material, and the remobilization of valuable nutrients. How autophagy regulates cell survival and death however, is still not well understood and should be a priority for future research.

## Methods

### Plant material and growth conditions

All experiments were performed on fine roots and leaves of *Populus trichocarpa* (Torr. & Gray) growing at an experimental field site at the Institute of Dendrology, Polish Academy of Sciences in Kórnik (52°14′40″N and 17°06′27″E).

Seeds were obtained from the FLORPAK Młynki Seeds Store, Poland. Seedlings were initially grown in a plant growth chamber (*Conviron GR96*) at 18 °C day/14 °C night and a 16 h day/8 h night photoperiod. After 3 months, plants were transferred into rhizotrons. The rhizotrons (50x30cm) were constructed of two transparent polycarbonate plates held 3 cm apart by thick-walled plastic tubing to provide sufficient growing space. The rhizotrons were placed in an underground chamber. They combine the controlled conditions of laboratory experiments with the advantages of a natural field setting. Waterlogging was avoided by providing a drainage hole in the bottom of each rhizotron. This permitted soil aeration and drainage of excess water. An automated system was used for the watering of individual plants. Plants were grown in rhizotrons consisting of clear-walled chambers filled with natural soil that allow shoots to grow above the soil surface. Rhizotrons were installed in a semi-open, foil greenhouse, to prevent flooding and heat stress. The rhizotrons provide the ability to collect root growth measurements over time without disturbing aboveground plant growth and without the need for destructive sampling of roots until deemed necessary based on the experimental design.

Senescent leaves were identified based on chlorophyll measurements (Fig. 1) and senescent roots were identified based on symptoms as defined by Comas et al. [50]. Additional data obtained on anatomy, cytology and a viability test were also taken into account when interpreting the collected data.

Samples were collected three times during a growth season. The first collection was considered as a control and was collected in early summer (July 7–15) when leaves and the root system were fully developed and functional. Control leaf samples were designated as LC and control fine root samples were designated as RC. The second group of leaf and root samples were harvested in early autumn (October 1–7) when chlorophyll levels in leaves had decreased by approximately 40% (Fig. 1) and when fine roots had changed in color from white to brown. The first stage of leaf senescence was designated as LS1 and the first stage of fine root senescence was designated as RS1. The third group of samples were harvested in the middle of autumn (November 2–9) when chlorophyll levels in leaves decreased by approximately 65% (Fig. 1) and fine roots were dark brown or black color. The second stage of leaf senescence was designated as LS2 and the second stage of fine root senescence was designated as RS2.

### Morphological studies

Photographic documentation of leaves was collected along with chlorophyll measurements to better illustrate the relationship between the two parameters during the senescence process. Chlorophyll levels were measured several times during the growth season using a CCM-200 plus Chlorophyll Content Meter (Opti-Sciences). Changes in the morphology of fine roots were examined several times during the growth season. This was done by removing the rhizotrons from the chamber and taking photos of the root systems, and immediately returning them back into the chamber. The same 30 plants were analyzed each time.

### Viability test using a fluorescein diacetate (FDA) staining assay

The viability of cells in fine roots and leaves was assessed with fluorescein diacetate (FDA)(Sigma). After harvesting, fine roots and leaves were cut into 35 μm thick cross-sections using a Leica VT1200S vibratome (Leica Biosystems, Nussloch, Germany). The sections were transferred to 100 μl of a diluted stock solution of FDA (stock solution 5 mg FDA in 1 ml of acetone, stock solution diluted 1:250 in Phosphate-buffered saline (PBS) (Sigma). After a 15 min incubation period at room temperature (RT), sections were rinsed three times in PBS buffer. Fluorescence was only observed in live cells due the conversion of non-fluorescent fluorescein diacetate into fluorescein. Fluorescence was induced by exposure to a wavelength of 470 nm (blue excitation and green fluorescence) under an Axioscope A1 microscope (Zeiss, Jena, Germany). Fluorescence images were digitally captured.

### Anatomical studies

The harvested samples of fine roots and leaves were immediately fixed in a mix 2% (*v*/v) glutaraldehyde (pH 6.8; Polysciences, Warrington, USA) and 2% (v/v) formaldehyde (pH 6.8; Polysciences, Warrington, USA). After an overnight incubation in fixative solution, the samples were rinsed three times with a cacodylate buffer (0.05 M; pH 6.8; Polysciences) and then dehydrated in a graded ethanol series (10–100%, v/v). Subsequently, the samples were incubated in a series of ethanol:Technovit 7100 resin mixture (Heraeus Kulzer, Wehrheim, Germany) with ratios of 3:1, 1:1, 1:3, and finally in pure Technovit 7100 resin. Cross-sections were cut with a Leica RM2265 Fully Automated Rotary microtome (Leica-Reichert, Bensheim, Germany) at a thickness of 10 μm. The cross-sections were stained with 1% (m/v) aniline blue and examined under a light microscope (Axioscope A1, Carl Zeiss, Jena, Germany).

### Cytological studies

For cytological studies, the fragments of fine roots and leaves were fixed in 2% (v/v) glutaraldehyde (pH 6.8; Polysciences, Warrington, USA) and 2% formaldehyde (v/v) (pH 6.8; Polysciences, Warrington, USA) at 4 °C overnight. Subsequently, the samples were rinsed three times with a cacodylate buffer (0.05 M; pH 6.8, Polysciences) and postfixed in 1% (v/v) osmium tetroxide (Polysciences) at RT for 2 h. The double fixed material was counterstained for 1 h with 2% uranyl acetate (Polysciences) and embedded in low viscosity resin using the method described by Zenkteler and Bagniewska Zadworna [79]. Ultrathin sections (70 nm) were cut on a Leica EM UC7 (Leica-Reichert, Bensheim, Germany) ultramicrotome using a diamond knife and cut sections were collected on formvar-coated copper grids. The sections were stained with uranyl acetate and lead citrate, and examined with a Hitachi HT7700 transmission electron microscope (Hitachi, Tokyo, Japan) operating at an accelerating voltage of 80 kV.

### Protein extraction, gel electrophoresis, and western blot analysis

Total protein was extracted from the collected samples according to the method described by Szuba et al. [80], which is based on phenol extraction. After extraction, proteins were solubilized in a buffer containing 7 M urea, 2 M thiourea, 40 mM dithiothreitol (DTT), 0.5% carrier ampholytes, and 4% CHAPS. Protein concentration was measured with a 2-D Quant Kit (GE Healthcare, Piscataway, USA). Proteins were separated by SDS-PAGE on 12% polyacrylamide gels, with an equal amount of protein (20 μg) in each lane. The western blot analysis was performed according to the method described by Kalemba and Litkowiec [81]. A primary antibody - anti-ATG8 (Agrisera) was diluted 1:1000. The presence of reactive protein was visualized on a membrane using an alkaline phosphate substrate (5-bromo-4-chloro-3-indolyl phosphate/nitro blue tetrazolium) (Sigma Aldrich, St. Louis, USA).

### RT-qPCR analysis of gene expression

RNA isolation was performed with a Ribospin Plant kit (GeneAll Biotechnology Co., Ltd., Korea) according to the manufacturer’s recommendations. RNA was suspended in nuclease free water and stored at − 80 °C. cDNA synthesis was performed using a High Capacity cDNA Reverse Transcription kit (Applied Biosystems, Thermo Fisher Scientific Inc., USA) following the protocol supplied by the manufacturer. Reverse transcription – quantitative PCR (RT-qPCR) was carried out using a SYBR Green Master Mix kit (Applied Biosystems, Thermo Fisher Scientific Inc., USA). All analyses of gene expression by RT-qPCR utilized three technical replicates from three biological replicates of each experimental variant. Analyses were conducted in 96-well plates in a CFX96 Touch Real-Time PCR Detection System (Bio-Rad Laboratories, Inc., USA) utilizing the following amplification program: denaturation by a hot start at 95 °C for 10 min, followed by 40 cycles of a two-step program (denaturation at 95 °C for 15 s and annealing/extension at 60 °C for 1 min). Primers used in this study were designed using Primer3 software (The Whitehead Institute for Biomedical Research, Cambridge, MD, USA). The sequences of the primer pairs are listed in Table 1. Several reference genes (such as: *GADPH*, *Actin*, *18S rRNA*, *ß-Tubulin*, *PKFE*, *EF1a*, *NADH*, and *Ubiquitin*) were utilized. *ß-Tubulin*, *GAPDH*, and *Ubiquitin* were selected as housekeeping genes and for normalization of expression values because they exhibited the lowest sample to sample variation and high stable expression in all samples types and time points. Data analyses were performed according to the method described by Bagniewska-Zadworna and Stelmasik [82]. The average cycle threshold (Ct) values of the reference genes were subtracted from the corresponding Ct value of each gene to obtain a ^∆^Ct value, and the relative expression levels were calculated using the ^∆∆^Ct method.

**Table 1:** List of primer sequences used for RT-qPCR analyses

Gene	Primer Sequences
*ATG7*	F - 5’-GGAATCGAATTCCTGCTTCA-3’ R - 5’-TGTCTCATCATCCCAGTCCA-3’
*ATG8c*	F - 5’-TGCCTGTGTTACGGATCTTG-3’ R - 5’-ACCCCAAATGTGTTCTCACC-3’
*ATG8d*	F – 5’-GCCAACAGTGAGATCAGCAG-3’ R – 5’-GGGACTTTGTGAGGTGTGCT-3’
*ATG8g*	F - 5’-CGTTGCCTCAAACAGCAAGT -3’ R – 5’-AGAAAGGATGATACAGCTTAGCCA-3’
*ATG8h*	F - 5’-TAGAGAGGTGGTTGGGTGCT-3’ R – 5’-CCTGCTTCTGACCCTTCTTG-3’
*ATG11*	F- 5’- AGAGCTGCTTGACAAGTACCCA-3’ R- 5’-CTTTCCTTGTTTGCCTGCTTCT-3’
*ATG18*	F - 5’-GACAATGACGAGCCAGGATT-3’ R – 5’- AGAGTTCGAGTGGCTGGAGA-3’
*ß-TUBULIN*	F – 5’-TTCTCCTGAACATGGCAGTG-3’ R - 5’-CCACACAACGTGAAATCCAG-3’
*GAPDH*	F - 5’-CAATGAATGGGGCTACAGGT-3’ R – 5’-CATGAATCAGCTGCACATCC-3’
*UBIQUITIN*	F - 5’-AGGAACGCGTTGAGGAGAAG -3’ R – 5’-TATAABCAAAAACCGCCCCTG -3’

*F* forward primer, *R* reverse primer

### Immunodetection of ATG8 using a tyramide signal amplification (TSA) assay

A tyramide signal amplification (TSA) technique was used to assess the localization of ATG8 protein due to its high level of sensitivity. The TSA technique is approximately 1,000× more sensitive than the standard protocol for immunolocalization.

Pieces of fine roots and leaves were fixed in 2% (v/v) glutaraldehyde (pH 6.8; Polysciences, Warrington, USA) and 2% (v/v) formaldehyde (pH 6.8; Polysciences, Warrington, USA) for 12 h and then rinsed three times in 1xPBS (Sigma) buffer. Immunolocalization in leaf samples utilized 32 μm thick sections, which were obtained using a Leica VT 1200S (Leica Biosystems, Nussloch, Germany) vibratome. Fine root samples were dehydrated in a graded ethanol series (10–100%) and then infiltrated and embedded in Paraplast Extra (melting point – 57.8 °C; Sigma, St Louis, MO, USA). Fine root sections (20 μm) were obtained using a Leica RM2265 (Leica Biosystems, Nussloch, Germany) microtome.

For immunolocalization, the material was incubated in 3% hydrogen peroxide solution for 1 h at RT to quench endogenous peroxidase activity. Subsequently, the material was rinsed three times in 0,01 M PBS buffer and blocked with 2% bovine serum albumin (BSA, Sigma) for 20 min. A primary ATG8 rabbit antibody (Agrisera) was used for immunolocalization of ATG8 proteins. The primary antibody was diluted 1:1000 in 0.2% BSA (Sigma) and the sectioned material was incubated with the primary antibody at 6 °C overnight. The material was rinsed five times in PBS buffer and then incubated with poly-HRP-conjugated secondary antibody (Thermo Fisher Scientific Inc., USA, attached to TSA Super Boost kit) for 1 h at 36 °C. The antibodies were rinsed from the samples five times with PBS and then the samples were exposed to a working solution of tyramide for 8 min at RT. The working solution of tyramide was prepared according to the manufacturer’s directions (Thermo Fisher Scientific Inc., USA). The reactions were arrested by the addition of 100 μl of a stop reagent (Thermo Fisher Scientific Inc., USA). After rinsing in PBS buffer, the sectioned samples were mounted in Prolong Gold (Life Technologies). Results of the immunolocalization assay were recorded with a Leica TCS SP5 confocal microscope (Leica Biosystems, Nussloch, Germany). Negative control reactions produced an undetectably low signal compared with the standard reactions (Additional file 1, Figure S1).

### Statistical analysis

Statistical analyses (ANOVA and Tukey’s test) were performed using Statistica 12.0 software (StatSoft Poland Inc., Tulsa, OH, USA).

## Additional file

Additional file 1: **Figure S1.** Comparison of ATG8 immunolocalization reactions with a negative control. Figure. 1a, b – The localization of ATG8 in senescence leaf. Fig. 1c, d – The negative control reaction performed omiting the primary antibody. (TIF 42774 kb)

## Abbreviations

ATG: Autophagy related genes
BSA: Bovine serum albumin
GFP: green fluorescent protein
LC: Control (green leaves)
LS1: First stage of senescence (yellowing leaves)
LS2: Second stage of senescence (yellow leaves)
PBS: Phosphate-buffered saline
PE: Phosphatidylethanolamine
RC: Control (white fine roots)
RCB: Rubisco-containing bodies
RFP: Red fluorescent protein
RS1: First stage of senescence (brown fine roots)
RS2: Second stage of senescence (dark brown or black fine roots)
RT: Room temperature
TSA: Tyramide signal amplification
WT: Wild type
