# Supplementary figures and images for: Autophagy counteracts instantaneous cell death during seasonal senescence of the fine roots and leaves in Populus trichocarpa
Source: BMC Plant Biol. 2018 Oct 29;18:260. doi: 10.1186/s12870-018-1439-6 (PMC6206944; doi:10.1186/s12870-018-1439-6)

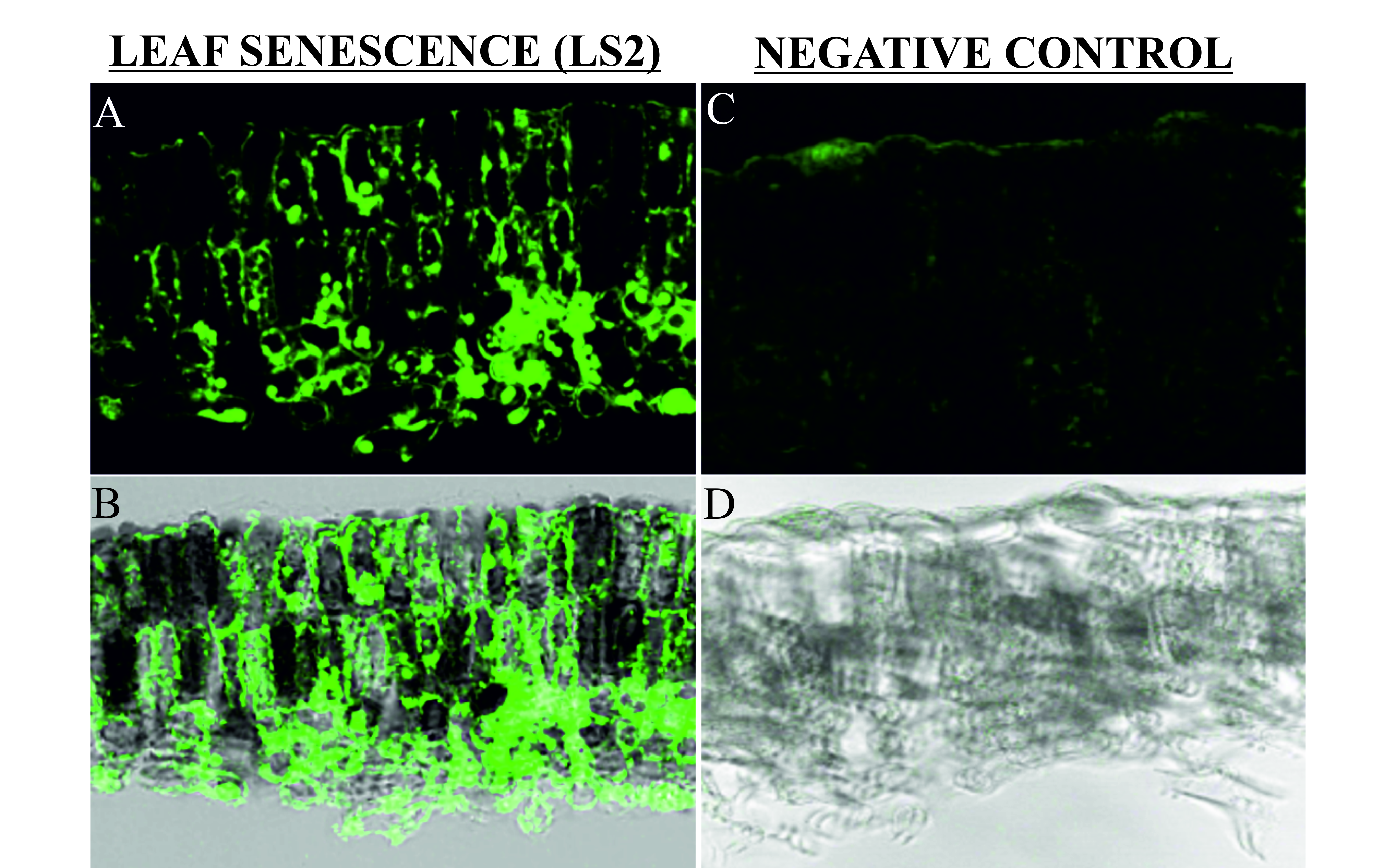

Supplement: Supplementary file 1 — Figure S1. Comparison of ATG8 immunolocalization reactions with a negative control. Figure. 1a, b – The localization of ATG8 in senescence leaf. Fig. 1c, d – The negative control reaction performed omiting the primary antibody. (TIF 42774 kb) [file 12870_2018_1439_MOESM1_ESM.tif]
